# The “Sunshine Vitamin” and Its Antioxidant Benefits for Enhancing Muscle Function

**DOI:** 10.3390/nu16142195

**Published:** 2024-07-10

**Authors:** Cristina Russo, Rosa Santangelo, Lucia Malaguarnera, Maria Stella Valle

**Affiliations:** 1 Section of Pathology, Department of Biomedical and Biotechnological Sciences, School of Medicine, University of Catania, 95123 Catania, Italy; lucmal@unict.it; 2 Department of Medicine and Health Sciences, University of Catania, Via Santa Sofia, 97, 95124 Catania, Italy; rosrac@yahoo.it; 3 Section of Physiology, Department of Biomedical and Biotechnological Sciences, University of Catania, 95123 Catania, Italy; maria.valle@unict.it

**Keywords:** calcitriol, oxidative stress, calcifediol, muscle homeostasis, muscular dysfunction, public health

## Abstract

Pathological states marked by oxidative stress and systemic inflammation frequently compromise the functional capacity of muscular cells. This progressive decline in muscle mass and tone can significantly hamper the patient’s motor abilities, impeding even the most basic physical tasks. Muscle dysfunction can lead to metabolic disorders and severe muscle wasting, which, in turn, can potentially progress to sarcopenia. The functionality of skeletal muscle is profoundly influenced by factors such as environmental, nutritional, physical, and genetic components. A well-balanced diet, rich in proteins and vitamins, alongside an active lifestyle, plays a crucial role in fortifying tissues and mitigating general weakness and pathological conditions. Vitamin D, exerting antioxidant effects, is essential for skeletal muscle. Epidemiological evidence underscores a global prevalence of vitamin D deficiency, which induces oxidative harm, mitochondrial dysfunction, reduced adenosine triphosphate production, and impaired muscle function. This review explores the intricate molecular mechanisms through which vitamin D modulates oxidative stress and its consequent effects on muscle function. The aim is to evaluate if vitamin D supplementation in conditions involving oxidative stress and inflammation could prevent decline and promote or maintain muscle function effectively.

## 1. Introduction

Skeletal muscle supports essential physiological processes ranging from movement to metabolism and respiration [1]. Muscle tissue has the function of protein storage with reserve function; if necessary, it can supply amino acids for energy production to other organs and tissues. Additionally, it influences the metabolism of lipids and is involved in the absorption and conservation of insulin-dependent glucose. In response to stress or nutrient deficiency, the modulation of muscular mass, metabolic needs, and muscle fiber composition become crucial to maintain the metabolic balance needed by other body organs governing physiological equilibrium [2]. Moreover, skeletal muscle exerts systemic effects by releasing cytokines and myokines. Considering the fundamental importance of muscular well-being in maintaining overall body stable equilibrium, preserving optimal skeletal muscle mass is linked to reduced mortality [3]. In pathological contexts, excessive catabolism provokes muscle loss and impairs skeletal muscle elasticity. The depletion of muscle metabolic stores shifts myokine regulation, and muscular fiber conformation facilitates the onset and progression of various diseases [4]. Muscle mass decline is not exclusive to advanced age [5]. It is intricately linked to co-morbidities in numerous pathological conditions such as lung, kidney, and heart failure, diabetes, autoimmune disorders, tumors, infectious, and neurodegenerative diseases [6,7,8]. Consequently, conditions marked by systemic inflammation and oxidative stress detrimentally impact muscle function by eliciting alterations in enzyme and mitochondrial activities [9], with resulting metabolic dysfunctions diminishing life quality. Skeletal muscle dysfunction in many instances progresses to sarcopenia or cachexia, both entailing elevated morbidity and mortality risks [1]. Normal concentrations of reactive oxygen species (ROS) and reactive nitrogen species (RNS) are fundamental for physiological cellular process regulation, including cell signaling activation, division, growth, regeneration, and cell death. Excessive oxidative stress becomes a predominant factor in causing harm to skeletal muscle. The intricate equilibrium between protein synthesis and degradation shapes the protein pool in the skeletal muscle. The energy status within muscle cells serves as a pivotal checkpoint governing the balance between hypertrophy and protein degradation, particularly during periods of energy stress, as it facilitates the provision of alternative energy sources required for adenosine triphosphate ATP production [10]. The impact of ROS/RNS disrupts Na/K-ATPase activity, calcium (Ca^2+^) handling within myofibrils, and actin–myosin interactions, leading to decreased muscle strength. Recent insights underscore correlations between mitochondrial aberrant morphology, dysfunction, and the shutdown of nuclear programs mediated by ROS and mitochondrial-derived metabolites via regressive signaling, governing muscle mass [11]. During muscle wasting, mitochondrial efficiency and consequently energy output diminish [11]. The functionality of skeletal muscle tissue is profoundly affected by dietary, environmental, physical, and hereditary factors. In vitro investigations have elucidated vitamin D’s determinative involvement in skeletal muscle function and its link to pain and muscle weakness [12]. Among its biological functions, vitamin D exhibits antioxidant capabilities. Vitamin D deficiency (VDD) is implicated in mitochondrial impairment, ATP reduction, and oxidative injury, which induces muscle atrophy and subsequent functional deterioration [8]. Notably, vitamin D appears efficacious in ameliorating functional impairments and muscle weakness [13]. This review delves into the molecular processes mediating vitamin D attenuation of oxidative stress and its impact on muscle function, aiming to ascertain the potentiality of vitamin D supplementation (VDS) in preventing muscle weakness.

## 2. Understanding the Biological Influence of Vitamin D on Muscle Performance

**Figure 1:**
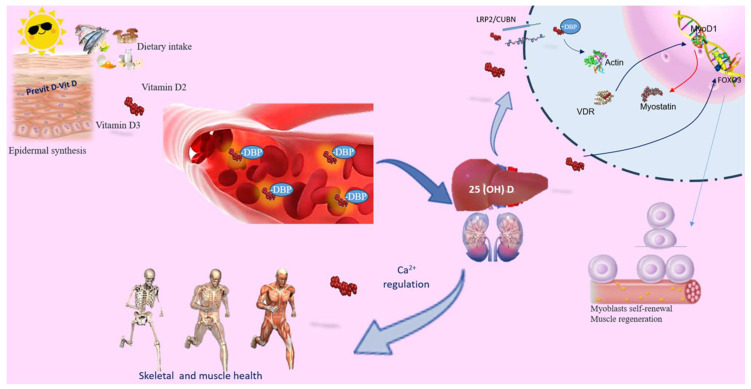
Vitamin D’s role in skeletal muscle. Vitamin D can be acquired through skin synthesis or from diet. Both vitamin D3 and D2 undergo the same metabolic processes to produce their active forms. While the primary role of vitamin D is to regulate calcium levels and ensure skeletal and muscle health, it also serves as a powerful immunoregulator, influencing the inflammatory response, muscle damage, and aerobic capacity. Circulating 25(OH)D binds to the carrier protein DBP. PTH promotes renal Ca^2+^ retention and activates the synthesis of active vitamin D, which, in conjunction with the vitamin D receptor (VDR), facilitates Ca^2+^ and phosphate absorption. Vitamin D deficiency (VDD) or inadequate sun exposure can elevate PTH levels, leading to skeletal fragility. In skeletal muscle, the 25(OH)D-DBP complex is transported into target cells through the LRP2/CUBN transmembrane complex. Inside the cell, the D-DBP complex associates with cytoplasmic actin. 1,25(OH)2D triggers the expression of protein 1, affecting MyoD1 activation. Vitamin D also regulates the FOXO3 signaling pathways, enhancing myoblast self-renewal. VDR expression in skeletal muscle promotes muscle protein synthesis, is crucial for maintaining muscle mass, and aids in muscle regeneration. Abbreviations in alphabetical order: 1,25(OH)2D3—calcitriol; FOXO—forkhead family of transcription factors; LRP2/CUBN—megalin–cubilin transmembrane complex; MyoD1—myogenic determination factor 1; PTH—parathyroid hormone; DBP—vitamin D binding protein; vitamin D receptor (VDR).

Calciferol, commonly known as vitamin D, exists in two active forms: D3 cholecalciferol (D3) and ergocalciferol (D2). It can be synthesized epidermally or absorbed from dietary intake. Skin synthesis occurs when UVB rays catalyze the conversion of 7-dehydrocholesterol (7DHC) into vitamin D3, while vitamin D2 is sourced from plant ergosterol. Vitamin D primarily regulates Ca^2+^ levels and supports skeletal health, with deficiencies linked to skeletal diseases including rickets and osteoporosis [14]. Additionally, vitamin D3 serves as a potent immunoregulator with unique impacts on inflammation, muscle damage, and aerobic capacity. Recent findings suggest its involvement in skeletal muscle regeneration [13]. Notably, the vitamin D mechanism has a broader distribution in precursor cells than in adult skeletal muscle [8,13] (Figure 1). Both vitamin D3 and D2 undergo a shared metabolic pathway to synthesize their active forms. Initially, they undergo conversion to 25-hydroxyvitamin D (25(OH)D; calcidiol) in the liver by 25-hydroxylase (CYP2R1). Calcidiol, the main circulating form of vitamin D, is utilized in clinical assessments. Around 85% of circulating 25(OH)D is bound to vitamin D binding protein (DBP), conducted to the kidney where it undergoes hydroxylation by 1α-hydroxylase (CYP27B1) to produce the biologically active metabolite known as 1α,25-dihydroxyvitamin D (1,25(OH)2D or calcitriol). Interestingly, CYP27B1 is also expressed in tissues like muscle, enabling local conversion of inactive to active vitamin D [15]. Moreover, within skeletal muscle cells, 25(OH)D bound to DBP is transported via the megalin–cubilin transmembrane complex (LRP2/CUBN). Inside cells, the D-DBP complex associates with cytoplasmic actin. 1,25(OH)2D stimulates the expression of protein 1, which triggers activation of myogenic determination factor 1 (MyoD1) and subsequent inhibition of myostatin. Moreover, vitamin D influences forkhead box O (FOXO) 3 and Notch signaling pathways, enhancing myoblast self-renewal and supporting the population of satellite stem cell (Figure 1) [8]. The biological actions of vitamin D are mediated through its nuclear receptor, known as the vitamin D receptor (VDR), found in muscle cells, highlighting its significant impact on skeletal muscle function [16]. The activation of vitamin D prompts the induction of VDR in satellite cells during muscle regeneration. This binding enhances intracellular phosphate absorption, essential for muscle contractility support [8]. Moreover, with 1,25(OH)2D addition in myoblasts, there is an augmentation in VDR expression, inhibiting cell growth and facilitating muscle cell differentiation [17]. Following muscle damage, VDR and CYP27B1 expression elevate significantly, indicating their function in muscle regeneration. Skeletal muscle harbors satellite cells crucial for regeneration post-injury. These cells undergo asymmetric division, maintaining the satellite cell pool while ensuring cellular differentiation progress [18]. Furthermore, the presence of VDR in muscle and satellite cells indicates that vitamin D may directly contribute to muscle regeneration [19]. Low levels of vitamin D are linked to muscle pain, decreased muscle mass, weakness, and an increased risk of sarcopenia. However, the exact involvement of vitamin D in maintaining skeletal muscle health remains incompletely understood.

## 3. Exploring the Role of Oxidative Stress on Muscle Weakness

Oxidative stress arises when ROS/RNS production outpaces the antioxidant defense systems, resulting in molecular and cellular damage that compromises tissue function. An excess of ROS/RNS can originate from multiple sources, such as changes in cellular metabolism, exposure to environmental pollutants, and poor lifestyle habits like cigarette smoking. These oxidative stressors detrimentally impact biological molecules and cell gene expression. Typically, compensatory antioxidant mechanisms are activated to restore redox balance [20]. ROS are critical components in redox signaling pathways, acting as key regulators of multiple intracellular responses. The effects of ROS, whether beneficial or harmful, are determined by the specific type, their concentration levels, and their production sites. At moderate concentrations, ROS mediate essential cellular functions, including stimulation–contraction coupling, cell differentiation, and proliferation. However, elevated ROS levels can disrupt intracellular molecular structures and functions. ROS compromise genomic DNA integrity, induce modifications or deactivate proteins through enzymatic processes, and perturb intracellular lipids by initiating lipid peroxidation [21]. For instance, the superoxide anion (O^2−^) can react with nitric oxide (NO) to deactivate NO, leading to peroxynitrite (ONOO) generation and endothelial dysfunction [22]. This reaction occurs not only in oxygen (O^2^) and NO-rich environments but also when antioxidant defenses are diminished. These harmful processes trigger muscle cell dysfunction, cell death, reduced contractility, fibrosis, hypertrophy, impaired muscle remodeling, and lastly, reduced performance. Low-grade inflammation is the primary contributor to oxidative stress, prevalent in various pathological conditions. Factors other than aging that contribute to the onset of oxidative stress include conditions such as cancer, heart disease, autoimmune disorders, and neurodegenerative diseases [23]. Notably, skeletal muscle is susceptible to low-grade inflammation [24]. Most markers of inflammation, including interleukin 6 (IL-6), soluble tumor necrosis factor-alpha (TNFα), and C-reactive protein (CRP), along with ROS generated within the local inflammatory environment, circulate throughout the body, activating inflammatory cells. This initiates a harmful cycle, leading to additional release of pro-inflammatory agents like TNFα, IL-1β, and ROS [25].

**Figure 2:**
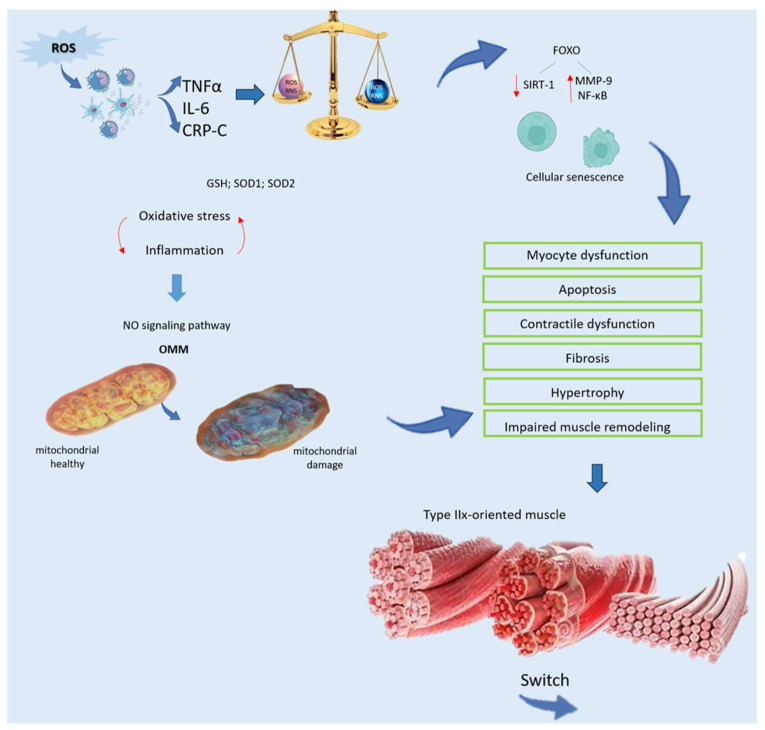
Relationship between oxidative stress and weakness of muscle. ROS generated from localized inflammation activate immune cells, initiating a harmful cycle characterized by the release of pro-inflammatory mediators, such as TNFα, IL-6, and CRP. Despite the presence of an impaired antioxidant system, including decreased levels of GSH and SOD, oxidative damage remains unchecked. Furthermore, inflammation disrupts mitochondrial function in muscle through the NO signaling pathway, triggering cell death via OMM permeabilization. Oxidative stress accelerates cellular senescence by activating FOXO and diminishing SIRT-1, leading to heightened MMP-9 and NF-κB activity. This escalated oxidative stress precipitates myocyte dysfunction and apoptosis, resulting in contractile dysfunction, fibrosis, hypertrophy, and impaired muscle remodeling. Additionally, there is a transition towards a type-IIx-oriented muscle phenotype with compromised oxygen distribution and utilization, ultimately impairing functionality. Abbreviations in alphabetical order: CRP—C reactive protein; FOXO—forkhead family of transcription factors; GSH—glutathione peroxidase; IL-6—Interleukin 6; MMP-9—matrix metallopeptidase 9; NF-κB—nuclear factor kappa-light-chain-enhancer of activated B cells; NO—nitrogen monoxide; OMM—outer mitochondrial membrane; RNS—reactive nitrogen species; ROS—reactive oxygen species; SIRT-1—sirtuin-1; SOD—superoxide dismutase; TNFα—tumor receptor necrosis factor alpha.

ROS are generated not only in response to inflammation, cellular responses to bacterial infections, or cytokine-mediated mechanisms as part of cellular defense [26], but also originate from biological processes such as mitochondrial oxidative metabolism, where ROS are produced as byproducts (Figure 2). Initially, O_2_ is produced, quickly transformed into hydrogen peroxide (H_2_O_2_) by superoxide dismutase (SOD), and ultimately converted into water by catalase or glutathione peroxidase [27]. Inflammation can greatly influence mitochondrial muscle function through the NO signaling pathway. Elevated production of nitric oxide by inducible nitric oxide synthase (iNOS) markedly disrupts the electron transport chain, elevates oxidative stress, and initiates apoptosis through permeabilization of the outer mitochondrial membrane (OMM) [28] (Figure 2). Inside cells, enzymes that produce ROS comprise membrane-bound NADPH oxidase (NOX), xanthine/xanthine oxidase, and the myeloperoxidase system derived from neutrophils (MPO), catalyzing chloride oxidation and generating hazardous hypochlorous acid [29]. Typically, intracellular antioxidant enzymes in healthy tissues thwart ROS formation, diminishing their detrimental cellular impacts. Among these enzymes are glutathione (GSH) peroxidase and SOD, comprising copper–zinc-SOD and manganese-SOD (SOD1 and SOD2, respectively) [30]. GSH, a crucial redox molecule, counteracts ROS toxicity, with the reduced-to-oxidized-GSH ratio (GSH/GSSG) being pivotal for cellular protection against oxidative damage [31]. The ratio of GSH/GSSG, assessed following cellular stimulation, reflects oxidative stress status in biological systems. Changes in this ratio are associated with dysfunctions such as inflammation, autoimmune diseases, sepsis, apoptosis, aging, and cancer. Oxidative stress can trigger cell aging through FOXO transcription factors, which, in turn, reduce the activity and expression of sirtuin-1 (SIRT-1). This is associated with elevated acetylation of MMP-9 and NF-κB [8]. NF-κB, in particular, modulates the expression of immune response genes such as IL-6, IL-8, IL-1β, TNF-α, and several adhesion molecules [32], while also serving as a key regulator of cell growth, specialization, and angiogenesis. In addition, numerous kinases influence oxidative signals by activating NF-κB [33]. Intriguingly, oxidizing/reducing agents inhibit/improve NF-κB DNA binding, respectively. Among antioxidants, thioredoxins exhibit opposite effects on NF-κB depending on their cellular location. Specifically, in the nucleus, thioredoxins facilitate NF-κB DNA binding, whereas in the cytoplasm, they impede IκB degradation and NF-κB activation [34]. Additionally, ROS activate the PI3K-mTOR pathway, promoting microRNA-34a upregulation, which inhibits SIRT-1 [35]. Collectively, oxidative stress profoundly influences peripheral tissue functions, including skeletal muscle tissue [8]. Increased ROS production modifies protein and lipid structures and releases both pro-inflammatory and anti-inflammatory cytokines, culminating in muscle wasting and tissue deterioration [36]. Elevated oxidative stress in skeletal muscle triggers a transition towards a type IIx muscle phenotype characterized by decreased oxygen utilization [8] (Figure 2).

## 4. Unveiling the Molecular Pathways of Vitamin D in Musculoskeletal Balance

**Figure 3:**
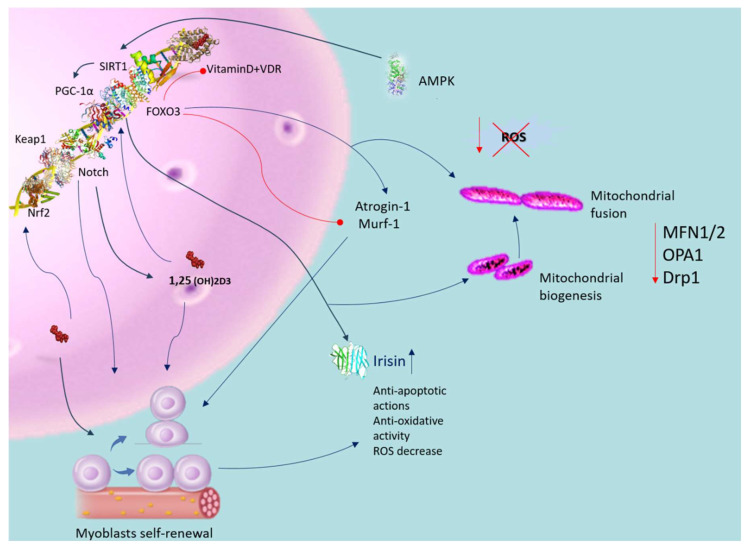
Antioxidative role of vitamin D in muscle dysfunction. Blue arrow indicates activation; red arrow indicates inhibition. Vitamin D activates the VDR in satellite cells, enhancing their self-renewal, proliferation, and differentiation capabilities. Activation of the VDR also mitigates oxidative stress, promoting mitochondrial biogenesis and fusion while reducing oxidative damage and dysfunction. This process improves mitochondrial network structure through the regulation of MFN1/2, OPA1, and Drp1 expression. Vitamin D positively influences Sirt1 activity and enhances mitochondrial function. Supplementation with vitamin D activates Sirt1 and AMPK in skeletal muscle cells. Vitamin D further increases the expression of irisin precursors in muscle cells, contingent upon intact Sirt1 expression. Both AMPK and Sirt1 regulate PGC-1α activation and transcription, influencing irisin secretion in skeletal muscle cells. Vitamin D upregulates FOXO1 protein and suppresses atrogin-1 and MuRF1 expression. Additionally, VDS triggers VDR and induces the Nrf2-Keap1 antioxidant pathway. Abbreviations in alphabetical order: AMP—5′ AMP-activated protein kinase; 1,25(OH)2D3—calcitriol; Drp1—dynamin-related protein 1; IL-1β—interleukin-1 beta; MFN1/2—mitofusin; OPA—mitochondrial dynamin like GTPase; PGC-1α—peroxisome proliferator-activated receptor-gamma coactivator; PTH—parathyroid hormone; ROS—reactive oxygen species; SIRT-1—sirtuin-1; SOD—superoxide dis-mutase; TNFα—tumor receptor necrosis factor alpha; VDR—vitamin D receptor.

To reveal the molecular pathways of vitamin D in musculoskeletal balance, it is central to know the role of parathyroid hormone and its relationship with vitamin D. Both are important hormones for the regulation of calcium. In particular, PTH promotes the synthesis of active vitamin D, which enhances the absorption Ca^2+^ and phosphate by interacting with the VDR. While vitamin D primarily influences Ca^2+^ levels rather than PTH directly, deficiency or insufficient sun exposure can elevate PTH levels. Increased PTH levels can cause bone degradation, leading to skeletal fragility [37]. Research indicates that vitamin D treatment in primary hyperparathyroidism (PHPT) alongside vitamin D deficiency enhances irisin levels in the bloodstream, thereby promoting better muscle cell development [38]. A recent observation has unveiled a connection between vitamin D and irisin, both crucial for regulating musculoskeletal function and energetic balance [39]. Both compounds play crucial roles in regulating the musculoskeletal system and maintaining energy balance (Figure 3). Mainly produced in skeletal muscles, irisin contributes positively to human health. It is activated through the γ-receptor and reliant on PGC-1α, which influences irisin release in skeletal muscle cells and controls mitochondrial content, crucial for the process of brown adipose tissue formation [40,41]. Moreover, PGC-1α enhances the irisin precursor, FNDC5, expression [42], impacting macrophages and adipocytes activity in inflammatory responses [43]. Irisin demonstrates antioxidative and antiapoptotic characteristics, enhancing the formation of antioxidant enzymes and reducing ROS production [44]. Additionally, irisin promotes fatty acid oxidation [45] and limits lipid accumulation in adipocytes. Treatment with vitamin D in skeletal muscle cells stimulates FNDC5 expression, potentially elevating irisin levels, as indicated by recent preclinical research. Studies on rats with vitamin D deficiency have shown decreased irisin levels [46], while VDS alters FNDC5 gene expression in diabetic rat models [47]. Previous research has indicated a negative correlation between irisin and vitamin D, observed in patients with type 1 diabetes mellitus [48] and Charcot–Marie–Tooth disease [49]. Vitamin D treatment activates Sirt1 and AMPK in skeletal muscle cells [50], promoting irisin precursor expression only in the presence of intact Sirt1 expression (Figure 3). Sirt1, a NAD-dependent protein deacetylase [51], activates AMPK [50], crucial for muscle fiber oxidative capacity and mitochondrial biogenesis, ultimately influencing PGC-1α activation and transcription [8,13], as depicted in Figure 3.

## 5. Vitamin D Deficiency and Muscle Dysfunction

VDD is prevalent worldwide, marked by a decrease in 25-hydroxyvitamin D3 levels below 25 nmol/L [52]. This deficiency contributes to diminished ATP production, heightened ROS production, and impaired mitochondrial function [53]. Multiple studies have associated VDD with diminished muscle function, higher prevalence of muscle weakness, and muscle wasting [54]. Several factors, including aging, exacerbate the risk of VDD due to inadequate nutrition and reduced capacity for vitamin D synthesis. Skeletal muscle expresses the vitamin D receptor (VDR), and alterations in its expression further compound VDD-associated muscle impairments [8,13]. VDD induces changes in muscle fiber types, typically resulting in atrophy of type II fibers, crucial for preventing falls [55]. Muscle biopsies from individuals with vitamin D deficiency frequently show muscle wasting, typically involving atrophy of type II fibers, widened interfibrillar spaces, and fat infiltration, resembling changes seen with aging [55]. Studies investigating VDS’s effects on muscle function have yielded varying results. Ceglia et al. [55] found that vitamin D did not change the relative proportion of type II fibers, and there was no significant difference in muscle extension power and physical performance. However, other studies have reported enhancements in muscle strength and performance, along with an increase in the diameter of type II fibers following vitamin D treatment. Further studies, performed on elderly subjects, corroborated these findings, demonstrating enhanced muscle strength, balance, and reduced fall risk with VDS [8,13,56]. Cellular models reveal that vitamin D treatment inhibits atrophy-related proteins like atrogin-1 and upregulates muscle-regulatory proteins such as muscle RING-finger protein-1 (MuRF1) and forkhead box O1 (FOXO1) [57]. In vivo studies suggest that VDD diminishes SIRT-1 activation, impairing muscle development and function, whereas VDS enhances these parameters [58] (Figure 3).

Intramuscular VDS supports muscle regeneration by enhancing VDR protein expression, crucial for the function of both type I and type II muscle fibers [19]. Genetic variations in the VDR gene may influence muscle function, particularly in older adults, where decreased VDR expression with age contributes to reduced muscle strength [8,13]. Reviews by Russo et al. [8] and Valle et al. [13] emphasize that VDR gene polymorphisms can impact muscle performance, though the specific interaction mechanisms remain unclear. Furthermore, these polymorphisms affect the role of vitamin D in age-related muscle dysfunction. Notably, age-related reduction in VDR expression contributes to diminished muscle strength [8,13]. Furthermore, studies using VDR knock-out mice reveal not only muscle weakness, fiber atrophy, and increased nuclear size but also disrupted patterns of expression for myogenic transcription factors [8].

## 6. The Antioxidative Capacity of Vitamin D in Muscle Dysfunction

Vitamin D plays a crucial role in cellular and tissue protection by mitigating oxidative stress, although its interaction with its receptor and ROS signaling is intricate [59]. As already mentioned above, vitamin D regulates Ca^2+^ homeostasis in skeletal muscle, a key element in muscle energy metabolism due to its involvement in the interaction between cytosol and mitochondria [60]. Dysfunctional mitochondria contribute to intracellular Ca^2+^ level increases, impacting cellular metabolic homeostasis [61]. VDD negatively influences protein synthesis and degradation, particularly affecting the ATP–ubiquitin-dependent proteolytic pathway, which is vitamin D dependent [62]. Hence, VDD might lead to inadequate levels of mitochondrial Ca^2+^, causing disruptions in cellular metabolic balance [63]. The metabolized form of 1α,25(OH)2D3, crucial for muscle regeneration and contraction regulation, becomes inadequate in VDD, leading to disruptions in muscle contraction kinetics and increased ROS-mediated cytotoxicity [8]. Clinical studies show that muscle wasting and weakness develop at low levels of 25-hydroxyvitamin D (<50 nmol/L) [64]. Within muscles, one of the factors contributing to waste stems from disproportionate rates of protein breakdown and synthesis [13]. VDD contributes to alterations in antioxidant enzyme activities, influencing nitrosative stress, lipid and protein peroxidation, and reducing antioxidant enzyme activity in skeletal muscle [8]. The C2C12 cell line treated with 1,25-dihydroxyvitamin D showed decreased production of ROS, reduced protein ubiquitination, decreased protein and lipid oxidation, impaired intracellular function, muscle breakdown, and atrophy. In contrast, in the paraspinal muscle, 1,25-dihydroxyvitamin D enhances the function of glutathione peroxidase (GPx), superoxide dismutase (SOD), and indicators of mitochondrial generation [62]. VDD subjects receiving 1α,25(OH)2D3 supplementation exhibit increased rates of mitochondrial oxidative phosphorylation, as demonstrated by Bhat and Ismail [64]. Moreover, in skeletal muscle cells, stimulation with 1α,25(OH)2D3 enhances the oxygen consumption rate (OCR) and ATP generation, as observed in the study by Ryan et al. [65]. The vitamin D status influences alterations in the mitochondrial dynamics of skeletal muscle, phosphorylation of pyruvate dehydrogenase, and expression of nuclear genes encoding mitochondrial proteins, thereby impacting skeletal muscle performance, according to findings by Seldeen et al. [66]. However, contradictory findings suggest that 1α,25(OH)2D3 does not induce an increase in OCR in mitochondria, implying a potential vitamin D receptor (VDR)-dependent effect [64]. Optimal levels of ROS are crucial for signal transduction following muscle injury. Excessive ROS production due to compromised antioxidant systems can lead to tissue muscle damage and compromised health. Increased body weight has been observed in animals treated with vitamin D hyper-exposure [8]. Experimental studies have demonstrated that vitamin D deprivation in mice for 1 year leads to decreased anaerobic capacity, lean mass, and gait instabilities, along with a susceptibility to smaller cross-sectional areas of fast-shrinking fibers and sarcopenia. Additionally, VDD mice show an increased expression of the atrogin-1 gene associated with atrophy and a different expression of mir-26a associated with muscle regulation compared to control mice [67]. Rats treated with vitamin D exhibit reduced oxidative stress and tissue impairment following exhaustive exercise [67]. Investigations suggest that vitamin D analogues can preserve skeletal muscle and cells under oxidative stress conditions, underscoring the essential work of vitamin D in mitochondrial function and oxidative stress regulation in skeletal muscle [19]. The mechanism by which vitamin D regulates oxidative stress may involve its effects on mitochondrial activity and dynamism. Nuclear factor erythroid 2-related factor 2 (Nrf2) is a critical transcription factor involved in antioxidant defense pathways [68]. The decrease in Nrf2 activity leads to the breakdown of the antioxidant defense system (Xiang et al., 2021) [69]. VDS activates VDR [70] and triggers the antioxidant Nrf2-Keap1 pathway [68] (see Figure 3).

## 7. Examining the Role of Mitochondrial Function in Muscle Weakness

Mitochondria exhibit a heightened sensitivity to fluctuations in reactive oxygen species (ROS) levels. Under conditions of cellular stress, this delicate balance is disrupted, leading to mitochondrial dysfunction and the activation of muscle autophagy and catabolic pathways [21]. Efficient mitochondrial function is crucial for maintaining skeletal myocyte homeostasis, given their heavy reliance on oxidative phosphorylation (OXPHOS) for energy production. However, the decline in cellular respiration compromises mitochondrial bioenergetics, alters OXPHOS, increases ROS production [71], and diminishes ATP synthesis. The ROS-induced opening of mitochondrial transition pores reduces β-nicotinamide adenine dinucleotide (NAD) reserves, leading to apoptosis. Furthermore, depletion of the mitochondrial fusion factor optical atrophy protein 1 (OPA1) disrupts mitochondrial integrity, exacerbating apoptosis [71]. Conversely, inhibition of fission proteins 1 (Fis1) or dynamin-related protein 1 (Drp1) attenuates mitochondrial fragmentation and apoptosis [11] (Figure 3). However, excessive fission stimulation can lead to mitochondrial dysfunction. Mitochondrial DNA (mtDNA) is particularly vulnerable to oxidative stress due to its lack of histones and introns, coupled with a relatively fragile repair system compared to nuclear DNA [72]. Oxidative-stress-induced mtDNA damage disrupts electron transport chain subunits, impairing OXPHOS, reducing ATP synthesis, and perpetuating ROS generation [73]. Subsequent muscle failure results in protein disaggregation and compromised antioxidant pathways, exacerbating mitochondrial impairment. Exposure to sub-cytotoxic doses of H_2_O_2_ suppresses Fis1, promoting elongated mitochondria production with increased ROS release [8]. Dysregulation of fusion and fission may preserve myocytes temporarily, but the accumulation of damaged mitochondria inhibits their clearance, exacerbating dysfunction. Mitochondrial damage intensifies ROS formation, driving inflammation and perpetuating the disease process [8,13]. The excessive ROS formation and reduced respiratory activity show a relationship with decreased physical activity induced by IFN-β. IFN-β-triggered ROS production in human myotubes can cause mitochondrial damage. Notably, heightened expression of both type I and type II IFN genes in the myocytes of diabetes mellitus patients correlates with the upregulation of genes involved in inflammatory responses and tissue repair [8,13]. Inflammation significantly impacts mitochondrial function, with NO having a prominent role in regulating biogenesis, O_2_ depletion, and redox homeostasis [74]. TNF-α-mediated induction of inducible nitric oxide synthase (iNOS) impairs mitochondrial function [72] by inhibiting the electron transport chain, promoting oxidant production, and inducing apoptosis [75]. TNF-α also induces apoptosis via the death receptor signaling pathway [76]. Moreover, elevated TNF-α levels downregulate the expression of PGC-1α, mitochondrial transcription factor A (TFAM), and nuclear respiratory factor 1 in C2C12 myoblasts, thereby inhibiting mitochondriogenesis [77] (Figure 2). Notably, in conditions such as chronic obstructive pulmonary disease (COPD), the inflammatory response significantly affects muscle oxidative capacity. This is demonstrated by alterations in citrate synthase activity, changes in PGC-1α expression, and reductions in type IIa oxidative muscle fibers [78]. Consequently, chronic low-grade inflammation and oxidative–antioxidant imbalance may contribute to cellular phenotype changes across various tissues, compromising overall organ and tissue homeostasis.

## 8. Modulating Mitochondrial Functionality through Vitamin D Regulation

As highlighted earlier, the upregulation of pro-inflammatory cytokines such as IL6, TNF-α, and plasma C-reactive protein levels can detrimentally impact mitochondrial muscle activity [79]. Inflammatory suppression of autophagy may exacerbate mitochondrial dysfunction [80]. Mitochondria are adept at releasing superoxide anions, and their formation is meticulously regulated by PGC-1α, which promotes mitochondrial biogenesis. This regulatory mechanism can encourage the transformation of muscle tissue into a fiber-like structure with metabolic traits that favor oxidation rather than glycolysis [81]. Mitochondria are skilled at releasing superoxide anions, and their biogenesis is strictly regulated by PGC-1α, a transcriptional coactivator recognized for managing oxidative stress and stimulating mitochondrial formation. This regulatory mechanism can facilitate the remodeling of muscle tissue toward a fiber-like structure with metabolic properties favoring oxidation over glycolysis [81]. Noteworthy, mitochondria are fundamental in the progression of atrophy [82]. Mitochondria, major contributors to superoxide anion release, are regulated by PGC-1α, a transcriptional coactivator crucial for mitochondrial biogenesis and oxidative stress regulation [81]. High PGC-1α levels inhibit the transcriptional activity of FOXO3a, which controls various atrophy-related genes, promoting muscle tissue remodeling towards a metabolically oxidative composition [82,83]. Conversely, FOXO factors inhibit cell cycle progression and activate apoptosis [84]. VDD has been shown to decrease PGC-1α and insulin-like growth factor 1 (IGF-1) via the VDR. C2C12 cell line study revealed that vitamin D treatment boosted VDR signaling and inhibited the nuclear expression, activity, and translocation of FOXO1. Notably, inhibition of FOXO1 activity decreased when the VDR was repressed, underscoring the crucial involvement of FOXO1 as a regulator of VDR signaling in skeletal muscle atrophy [85]. Moreover, Akt is recognized as a significant contributor to the progression of muscle atrophy [86]. Akt inhibits the action of FOXO3a by phosphorylating residues essential for its activity, thereby impairing its function toward target genes [86]. Akt-mediated phosphorylation prevents the nuclear translocation of FOXO3a, thereby inhibiting the expression of target genes associated with muscle atrophy, including F-box (MAFbx) and Murf proteins. The signaling pathway promoting MuRF1 and MAFbx expression is mediated by Src-ERK1/2-Akt-FOXO [87]. Furthermore, Akt modulates muscle synthesis via the mammalian target of rapamycin (mTOR). In a mouse skeletal myostatic tube experimental model, 1α,25(OH)2D3 induces the Akt/mTOR-dependent pathway, thereby facilitating protein synthesis activation [82]. Sirtuin-1 is a crucial regulator of biogenesis, inflammation, apoptosis, oxidative stress, cellular senescence, and mitochondrial activity. SIRT-1-catalyzed deacetylation facilitates FOXO activity and enhances its DNA binding affinity [88]. Vitamin D stimulates SIRT-1 expression, exerting positive effects on Sirt1 protein and mitochondrial activity [8] (see Figure 3). Moreover, the VDR influences FOXO protein function and selectively modulates SIRT-1, which, in turn, regulates VDR signaling [8]. The 1,25(OH)2D3-VDR complex in satellite cells and myocytes of the myocardium supports differentiation, cell proliferation, and self-renewal. Furthermore, VDR decreases oxidative stress and mitochondrial dysfunction and modulates MFN1/2, OPA1, and Drp1 expression, facilitating the mitochondrial networks renewal (Figure 3).

## 9. Conclusions

VDS may prevent decline and promote muscle function in oxidative stress and inflammation conditions. Recently, it has been evidenced that vitamin D shows neuroprotective properties, including the modulation of neuroinflammation, support of neuronal survival, and improvement of neurogenesis. These effects might indirectly benefit skeletal muscle health by improving the underlying neurological conditions that impair muscle function. Vitamin D is involved in several neurological disorders, such as multiple sclerosis, Alzheimer’s disease, Parkinson’s disease, and stroke [89], acting by inducing Th2 and regulatory T cells (Tregs), amplifying the innate immune system and regulating the adaptive immune system. In multiple sclerosis subjects, the vitamin D blood concentration has been related to the suppressive action and number of Tregs [90]. Several studies have shown that Tregs increase in patients with multiple sclerosis who have been supplemented with vitamin D [91]. According to many studies, vitamin D can influence the balance between inflammatory and anti-inflammatory mechanisms, potentially supporting the remyelination process. Diseases linked to low-grade inflammation and ROS/RNS concentration increase lead to muscle dysfunction, triggering cellular phenotypic fluctuations such as apoptosis and autophagy. Moreover, the anti-oxidative system decline, abnormal steroid synthesis, and mitochondrial damage accompanied the oxidative-stress-induced cascade. While the exact mechanism of oxidative stress is still under study, several signaling pathways have been identified, including the PI3K/AKT, MAPK, FOXO, and Nrf2/KEAP1 axes, as well as inflammatory pathways and mitophagy. Emerging observations claim how vitamin D can counteract muscle dysfunction by inhibiting oxidative stress and triggering signaling pathways. Nevertheless, the efficacy of VDS remains a subject of debate, with some studies yielding contradictory results. Several unresolved issues require further investigation regarding vitamin D. The precise molecular mechanisms of its antioxidant and neuroprotective effects remain incomplete, and optimal dosage and duration of supplementation need to be determined to maximize benefits while avoiding adverse effects. Additionally, the interaction between vitamin D and environmental and genetic factors in muscle health warrants further exploration. Future research should focus on elucidating the molecular pathways through which vitamin D influences oxidative stress and muscle function, identifying biomarkers to predict responses to vitamin D therapy and exploring its role in conditions like sarcopenia and muscular dystrophies. Investigating polymorphisms in the vitamin D receptor (VDR) is also crucial as genetic variations can significantly impact how individuals process and utilize vitamin D [92]. These variations can affect receptor binding affinity, vitamin D metabolism efficiency, and subsequent biological responses, making personalized approaches to prevention and treatment vital. In conclusion, further experimental investigations and clinical trials are essential to clarify vitamin D’s role in addressing oxidative stress and to validate its clinical utility. This review aims to stimulate ongoing dialogue and innovation, fostering new avenues for experimental studies and dietary interventions aimed at addressing oxidative-stress-related muscle disorders.

## Data Availability

Not applicable.
